# Neurogenomics: Challenges and opportunities for Ghana

**DOI:** 10.1016/j.atg.2015.06.002

**Published:** 2015-06-21

**Authors:** Thomas K. Karikari, Emmanuel Quansah

**Affiliations:** a Neuroscience, School of Life Sciences, University of Warwick, Coventry CV4 7AL, United Kingdom; b Midlands Integrative Biosciences Training Partnership, University of Warwick, Coventry CV4 7AL, United Kingdom; c Department of Molecular Biology and Biotechnology, School of Biological Science, University of Cape Coast, Cape Coast, Ghana; d Pharmacology, Faculty of Health and Life Sciences, De Montfort University, Leicester LE1 9BH, United Kingdom

**Keywords:** Neurogenomics, Neuroscience, Genomics, Big data, Bioinformatics, Scientific capacity, Africa, Ghana

## Abstract

The application of genomic tools and technologies has shown the potential to help improve healthcare and our understanding of disease mechanisms. While genomic tools are increasingly being applied to research on infectious diseases, malaria and neglected tropical diseases in Africa, an area that has seen little application of genomic approaches on this continent is neuroscience. In this article, we examined the prospects of developing neurogenomics research and its clinical use in Ghana, one of the African countries actively involved in genomics research. We noted that established international research funding sources and foundations in genomic research such as H3ABioNet nodes established at a couple of research centres in Ghana provide excellent platforms for extending the usage of genomic tools and techniques to neuroscience-related research areas. However, existing challenges such as the (i) lack of degree programmes in neuroscience, genomics and bioinformatics; (ii) low availability of infrastructure and appropriately-trained scientists; and (iii) lack of local research funding opportunities, need to be addressed. To promote and safeguard the long-term sustainability of neurogenomics research in the country, the impact of the existing challenges and possible ways of addressing them have been discussed.

## 1 Introduction

Genomic technologies are important resources in biomedical research and healthcare. Many healthcare delivery facilities, as well as research institutions and companies have adopted genomic tools and techniques to advance their activities (World Health Organization, 2002). In Africa, genomic research is gaining prominence particularly because of the wide coverage of sequencing technologies, making it possible to comprehensively characterise genetic variants in order to obtain further insights into disease mechanisms, as well as individual and population differences in disease susceptibility, resistance and drug responses (H3Africa Consortium, et al., 2014, Karikari, T.K. and Aleksic, J., 2015). A landmark study in this area is the African Genome Variation Project, which recently showed that genomic variations exist among some major African ethnic groups (Gurdasani et al., 2015). Furthermore, genome science has the potential to help improve drug discovery and development in Africa, through the identification and profiling of the efficacy of different drugs and drug candidates for treating diseases in people of different populations and genetic backgrounds (Karikari and Aleksic, 2015). This would also help to improve research into, and the development of, precision medicine.

In many parts of Africa, genome science has been applied to biomedical research areas such as infectious diseases, malaria, Human Immunodeficiency Virus/Acquired Immune Deficiency Syndrome (HIV/AIDS) and the neglected tropical diseases (Karikari, 2015a). However, one area that has seen little application of genomic approaches in Africa is neuroscience (Karikari and Aleksic, 2015). While genomic technologies have been employed on other continents to help provide further insights into the evolution, development, function and diseases of the nervous system, these areas of research are lacking in Africa. Yet, genome science provides many opportunities to develop neuroscience-related research on this continent. The ancestry of African populations is unique and often differs from those of other populations (Gomez et al., 2014). Moreover, the belief that modern humans originated from Africa suggests that improved studies of Africans would provide vital information that would advance neurological healthcare and neuroscience research (Gomez et al., 2014). In this article, we focus on the prospects of developing neurogenomics research and clinical use in Ghana, one of the African countries with active involvement in genome science research. We believe that the established foundations in genomic research in the country provide platforms to extend the use of genomic techniques to neuroscience-related research. We also discuss the challenges that would need to be addressed to enable this kind of transformation.

## 2 Opportunities for neurogenomics application in Ghana

Recent investments in genomics in Ghana have provided opportunities to advance this area of research. Important examples of these investments include the: (i) provision of research funds to Ghanaian investigators to study the genomic regulation of diseases such as stroke, tuberculosis, HIV/AIDS and malaria. These funds have been provided by, for example, the Human Heredity and Health in Africa (H3Africa; http://h3africa.org) project which is jointly funded by the National Institute of Health (NIH) in the United States and the Wellcome Trust (WT) in the United Kingdom as well as the Malaria Genetic Epidemiology Network (MalariaGEN; https://www.malariagen.net); and (ii) establishment of H3ABioNet nodes (centres of excellence) in Ghanaian research centres to deliver training and research in bioinformatics and genomics data analysis (H3Africa Consortium, et al., 2014, Karikari, T.K., 2015a). In addition, some international agencies (including the NIH and WT) often have funding programmes for genomics-related research in Africa that Ghana-based neuroscientists can take advantage of.

Ghana also has a considerable capacity in bioinformatics; scientists with this expertise can support genomics research design, data analysis and interpretation, as well as student training. In this regard, Ghanaian research facilities with bioinformatics and genomics capacities do organise training programmes (short courses and workshops) for scientists who would like to improve their knowledge in, and application of, these skills to advance their research activities. These programmes are helping to widen participation in genomics and bioinformatics in Ghana (Karikari, 2015a). Scientists working in neuroscience-related areas in Ghana could therefore collaborate with their colleagues who have in-depth knowledge of genomic techniques and data analysis to support research into neurological diseases and related disorders in the country.

Due to the rich biodiversity in Ghana, pharmacognosy research and the practise of herbal medicine are common in the country (van Andel et al., 2012). While pharmacognosy research usually focuses on ascertaining the potential usefulness of herbal products and other natural products as therapeutic agents against specific diseases, evaluation of the scientific basis of the apparent effectiveness of these products is often lacking (Mahomoodally, 2013). The adoption of neurogenomics would provide an opportunity to improve the clinical applicability of pharmacognosy research outcomes. This could be, for example, through the: (i) in-depth characterisation of the disease-modifying effects of bioactive compounds at the genomic and molecular levels; (ii) use of bioinformatics approaches to screen for drug potency against specific disease targets; (iii) possibility to support drug discovery and development by matching potential therapeutic agents with diseases that they would be effective against (Karikari and Aleksic, 2015).

Furthermore, the herbal medicine industry in Ghana has been growing, with many people relying on herbal products for their healthcare needs (van Andel et al., 2012). The use, and the effectiveness, of traditional medical approaches in treating neurological conditions have been documented in sub-Saharan Africa over several decades (Osuntokun, 1975). For this reason, the gradual integration of herbal medicine (or products) into the orthodox healthcare system in Ghana might be a good way to improve treatment outcomes. In this direction, initial attempts have been made to ascertain how the two systems could potentially work together. These attempts include the establishment of a herbal medicine degree programme and the integration of graduates from this programme into the mainstream national health service to support orthodox physicians in healthcare delivery (Adusi-Poku, Y., et al., 2009, Ministry of Health, Republic of Ghana,, 2007). While the aim behind this integration is to explore the feasibility of using herbal products in clinical care, the rigorous testing of these products for their safety and molecular mechanisms of action will be paramount. Genomic approaches will be useful in this regard.

Genetic and phenotypic diversity among African populations, including Ghanaian populations, is often higher than that of non-African populations (Campbell, M.C. and Tishkoff, S.A., 2008, Gomez, F., et al., 2014). Unfortunately, it remains largely unknown whether these diversities and population sub-structures among Africans affect susceptibility to and resistance from specific neurological diseases. The available evidence shows that the genetic basis of some neurological diseases (including specific neurodegenerative and motor neuron diseases) differ between specific African populations and also between African and non-African populations, suggesting that these diseases might progress in a population-specific manner (Karikari, T.K. and Aleksic, J., 2015, Quansah, E. and Karikari, T.K., 2015). However, the evidence based on the genetic and molecular basis of neurological diseases among Ghanaians is scanty. Improved use of neurogenomic tools might help to identify the genomic regulation of these diseases among people of Ghanaian descent, and how this might differ from other populations in Africa and elsewhere.

## 3 Challenges to neurogenomics use in Ghana

While there are prospects of using genomic techniques and tools to advance neuroscience research in Ghana, there are challenges that need to be addressed. These include the: (i) low numbers of appropriately-trained scientists and clinicians for clinical and experimental use of neurogenomics approaches, and (ii) lack of laboratory resources for this kind of research. Another challenge is the lack of degree programmes in genome science to train more scientists in this area. In this section, we will discuss how these weaknesses affect the adoption of neurogenomics. Other challenges such as issues regarding: ethical collection, use and sharing of genomics data; collaborative research; and a heavy burden of diseases with associated neurological complications have been previously discussed elsewhere (Karikari and Aleksic, 2015).

To begin with, experimental neuroscience research capacity is low in Ghana. In a recent evaluation of neuroscience research capacity in the country, we found that studies into the molecular, genetic and genomic basis of neurological health and disease were seriously lacking (unpublished data). We identified almost no research group leading this area of research in the country. However, before genomics can be applied to neuroscience, knowledge and expertise in experimental neuroscience is essential. The lack of scientists focused on bench science aspects of neuroscience (including molecular and genetic aspects) is a huge challenge that needs to be addressed.

Additionally, most higher education institutions in Ghana do not have the required laboratory resources and adequately-trained human resources to lead genomics research activities. It was recently reported that out of the many science-focused higher education and research institutions in the country, only a few (about twenty) have been involved in bioinformatics and genomics-related research work in the past decade (Karikari, 2015a). This inadequacy would negatively affect the adoption of genome science, particularly in areas such as student training and research application.

An important field where neurogenomics is used is clinical medicine (Boguski, M.S. and Jones, A.R., 2004, Tsuji, S., 2013). Genomic tools and techniques are used in the neurology clinic and research areas to better understand: (i) how diseases are genetically and/or genomically regulated; (ii) how these information can be used to help develop accurate diagnostic platforms for patients; (iii) how genomic regulation of diseases can be used to predict an individual's risk of developing a disease later in life; (iv) whether the risk of developing specific neurological diseases is more severe among different populations and ethnic groups; and (v) how effective therapeutic agents can be developed (Boguski, M.S. and Jones, A.R., 2004, Tsuji, S., 2013). To be able to develop clinical applications of neurogenomics in Ghana, more clinicians should be trained to incorporate genomic testing in their diagnosis and treatment criteria. However, clinicians with expertise in neuroscience are limited in Ghana, leaving the potential use of clinical neurogenomics in doubt. It was recently reported that there are only two neurologists actively practising in Ghana (Drislane et al., 2014a). Since Ghana has a population of about 26 million people, this translates to a neurologist to 13 million Ghanaians. This ranks poorly against the World Health Organization's recommendation of one neurologist to 100,000 persons (Bower and Zenebe, 2005). In the absence of neurologists and other clinicians with expertise in neuroscience-related areas, non-specialised clinicians can be trained to facilitate the use of neurogenomics in Ghanaian health facilities. Recent initiatives such as the introduction of pro-rural medical residency programmes and short courses to train non-neurology specialist clinicians in specific areas of neurology are helping to improve the number of specialist clinicians as well as those with considerable know-how in neurology to support the clinical use of genomics (Cilia, R., 2013, Drislane, F.W., et al., 2014b). More initiatives of this kind will be required.

To ensure the sustainable supply of scientists with expertise in the design, implementation, analysis and reporting of neuroscience and genomic research, degree programmes that provide top-notch training in these areas are needed (Karikari, T.K., 2015a, Karikari, T.K., et al., 2015a). In Ghana, degree programmes in neuroscience, genome science, bioinformatics and computational biology (training in bioinformatics and computational biology is required for genomic data analysis and the development of computational tools and methods for this analysis) are nonexistent (Karikari, T.K., 2015a, Karikari, T.K., et al., 2015a, Karikari, T.K., et al., 2015b). This means that there is currently no local opportunity for students who are interested in these areas to pursue their dreams. While there are plans to establish some of these training programmes in the near future, it is important that emphasis is placed on the development of up-to-date curricula and the provision of training resources to ensure that trainees would obtain world-class education in these areas (Karikari, T.K., et al., 2015a, Quansah, E. and Karikari, T.K., 2015). Aside from the establishment of degree programmes for exclusive training in neuroscience and genomics, improved integration of computational biology training into existing life science curricula in the country would be vital in ensuring that more life scientists are trained to be able to generate, analyse, interpret and report on large computational data (Karikari, T.K., 2015b, Karikari, T.K., 2015c).

The lack of local funding for bioinformatics research, and genomic research in particular, is another impeding factor to the adoption and use of neurogenomics and related applications in Ghana (Karikari, 2015a). Although the cost of genomic sequencing technologies have reduced drastically in recent years, many Ghanaian laboratories will find it difficult to purchase these equipment and the associated software and hardware for the processing and analysis of genomic data (Karikari, 2015a). Presently, Ghana has no governmental agency that administers and awards competitive research grants directly to scientists (United Nations Conference on Trade and Development (UNCTAD), 2011). Also, the Government's spending on local scientific research is less than 1.0% of the country's gross domestic product (Karikari, 2015a; United Nations Conference on Trade and Development (UNCTAD), 2011). This negatively affects the amount and quality of research conducted in the country, since only a few scientists can succeed in securing funding from international donor agencies. The Government of Ghana, educational authorities, the private sector and other stakeholders should do more to support scientific research in the country.

Lastly, ensuring good public understanding of the processes, outcomes and significance of genomic research is an important step in widening participation in this area (Tindana et al., 2012). This is because genomic research has ethical, legal and social implications (ELSI) that need to be effectively addressed in all research activities (Caulfield et al., 2013). While considerable progress has been made in ensuring that societal concerns are catered for in genomics research conducted in Africa, existing problems such as low literacy rates and low access to healthcare among African populations pose challenges to ensure that the ELSI of genomics research in Ghana are always properly addressed (de Vries et al., 2015). In order to safeguard the long-term sustainability of neurogenomics research and applications in Ghana, more innovative approaches should be developed to help the public better understand the risks and benefits of their involvement in this area of research (Karikari and Aleksic, 2015).

## 4 Conclusion

Neurogenomics is a developing area at the intersection of neuroscience, genome science and bioinformatics. Neurogenomics presents opportunities to help advance biomedical research in Ghana, for example, through the provision of further insights into the molecular basis of neurological disease risk, protection and progression, as well as to improve the testing and screening for herbal medicinal products. However, the existing challenges such as the lack of degree programmes in neuroscience, genomics and bioinformatics, and the inadequate expertise and laboratory resources for research and clinical application of neurogenomics need to be addressed to enable this transformation. Importantly, improved funding and policy support, particularly from the Government of Ghana, for neurogenomics research would be important to attract and equip the best-trained scientists in this area.
